# The crucial role of the cerebellum in autism spectrum disorder: Neuroimaging, neurobiological, and anatomical insights

**DOI:** 10.1002/hsr2.2233

**Published:** 2024-07-03

**Authors:** Mohammad Shahangir Biswas, Suronjit Kumar Roy, Rubait Hasan, Md Moyen Uddin PK

**Affiliations:** ^1^ Department of Biochemistry and Biotechnology Khwaja Yunus Ali University Sirajganj Bangladesh; ^2^ Department of Public Health Daffodil International University Dhaka Bangladesh; ^3^ Institute of Biological Science Rajshahi University Motihar, Rajshahi Bangladesh

**Keywords:** ASD, behavioral disease, brain function, cerebellar dysfunction, cerebellum

## Abstract

**Background and Aims:**

Autism spectrum disorder (ASD) is a complex neurodevelopmental condition characterized by a wide range of symptoms and challenges. While ASD is primarily associated with atypical social and communicative behaviors, increasing research has pointed towards the involvement of various brain regions, including the cerebellum. This review article aims to provide a comprehensive overview of the role of cerebellar lobules in ASD, highlighting recent findings and potential therapeutic implications.

**Methods:**

Using published articles found in PubMed, Scopus, and Google Scholar, we extracted pertinent data to complete this review work. We have searched for terms including anatomical insights, neuroimaging, neurobiological, and autism spectrum disorder.

**Results:**

The intricate relationship between the cerebellum and other brain regions linked to ASD has been highlighted by neurobiological research, which has shown abnormalities in neurotransmitter systems and cerebellar circuitry. The relevance of the cerebellum in the pathophysiology of ASD has been further highlighted by anatomical studies that have revealed evidence of cerebellar abnormalities, including changes in volume, morphology, and connectivity.

**Conclusion:**

Thorough knowledge of the cerebellum's function in ASD may lead to new understandings of the underlying mechanisms of the condition and make it easier to create interventions and treatments that are more specifically targeted at treating cerebellar dysfunction in ASD patients.

## 1 INTRODUCTION

Autism spectrum disorder (ASD) is a heterogeneous neurodevelopmental condition characterized by impairments in social interaction, communication difficulties, and repetitive behaviors or restricted interests.^1^ Traditionally, research into the neurobiological basis of ASD has primarily focused on cortical regions, such as the prefrontal cortex and the amygdala. However, emerging evidence suggests that the cerebellum, traditionally associated with motor control, may play a crucial role in the pathophysiology of ASD. For decades, research on ASD has primarily focused on cortical regions of the brain, such as the prefrontal cortex and the anterior cingulate cortex, due to their roles in social cognition and executive function. However, recent advances in neuroimaging and neuroanatomy have highlighted the involvement of the cerebellum, a subcortical structure classically associated with motor control.

Traditionally, ASD research has predominantly focused on the cerebral cortex, particularly the frontal and temporal lobes, as key regions associated with the disorder. However, recent advances in neuroimaging and neuroanatomy have highlighted the role of the cerebellum in ASD. This review aims to provide an in‐depth analysis of the involvement of the cerebellum in ASD, drawing on both anatomical and functional perspectives, and discussing potential mechanisms underlying its contribution to the disorder. While the primary focus of ASD research has been on the cerebral cortex and its connectivity, growing evidence suggests that the cerebellum, long thought to be primarily involved in motor coordination, may also play a significant role in the pathophysiology of autism.^2^, ^3^

The cerebellum is a highly organized brain structure located in the posterior cranial fossa, traditionally associated with motor control and coordination. It consists of distinct lobules, each with specific functional properties.^4^ Recent neuroimaging studies have revealed that the cerebellum is not solely involved in motor functions but also plays a crucial role in cognitive and emotional processing.^5^ This expanded view of cerebellar function is essential in understanding its potential involvement in ASD.

Structural alterations in cerebellar lobules in ASD: Numerous neuroimaging studies have reported structural abnormalities in the cerebellum of individuals with ASD. These abnormalities often include differences in cerebellar lobule size, particularly in lobules VI and VII.^6^ Additionally, postmortem studies have highlighted reduced Purkinje cell density and disorganized cerebellar microarchitecture.^7^ These structural alterations may contribute to the motor and cognitive challenges observed in individuals with ASD.

Recent research has revealed that the cerebellum is involved in a wide range of cognitive and affective processes, including attention, language, working memory, and emotional regulation.^8^, ^9^ Given these expanded functions, it is reasonable to suspect that cerebellar abnormalities may contribute to the diverse symptoms observed in individuals with ASD.

The cerebellum, with its distinctive structure composed of lobules, has been implicated in various cognitive and affective functions beyond motor control.^10^ Recent neuroimaging and postmortem studies have provided insights into the specific involvement of certain cerebellar lobules in ASD, shedding light on the potential mechanisms underlying the disorder and opening new avenues for research and treatment.

Functional aberrations in cerebellar lobules in ASD: Functional neuroimaging studies have provided further insights into the cerebellar involvement in ASD. Altered activation patterns within cerebellar lobules during tasks related to social cognition, language processing, and motor coordination have been observed.^11^ Dysfunctional connectivity between the cerebellum and other brain regions, such as the prefrontal cortex and limbic system, has also been reported.^12^ These findings suggest that cerebellar dysfunction may contribute to the core symptoms of ASD.

The cerebellum is crucial for fine motor coordination, balance, and precise timing of movements.^13^ Furthermore, emerging research indicates that the cerebellum contributes to cognitive processes, including executive functions, working memory, and language.^14^ These cognitive functions are often impaired in individuals with ASD, further highlighting the potential significance of cerebellar dysfunction.

Purkinje cells are the principal neurons of the cerebellar cortex, sending inhibitory signals to the deep cerebellar nuclei. Granule cells are the most abundant neurons in the cerebellum, located in the granular layer. They receive input from mossy fibers and send excitatory projections to Purkinje cells through parallel fibers. Basket cells, stellate cells, and Golgi cells are inhibitory interneurons found in the molecular layer, regulating the activity of Purkinje cells. Deep cerebellar nuclei neurons are located within the white matter of the cerebellum and receive input from Purkinje cells and other sources, projecting output signals to various brain regions. Glial cells provide support and insulation to neurons. They include astrocytes, oligodendrocytes, and microglia, which play essential roles in maintaining the cerebellar microenvironment and supporting neuronal function.^15^

The development of the cerebellum involves the proliferation and migration of neuronal precursors. Neurogenesis primarily occurs during embryonic and early postnatal stages, guided by complex genetic and molecular mechanisms. The cerebellar cortex undergoes a process of lamination, where neurons migrate to form distinct layers: the molecular layer, Purkinje cell layer, and granular layer. This process is crucial for establishing the functional architecture of the cerebellum.^16^

During development, neurons in the cerebellum establish intricate synaptic connections. For instance, granule cells form synapses with mossy fibers, while Purkinje cells receive inputs from parallel fibers and climbing fibers. This synaptic refinement continues into early adulthood, shaping the functional circuitry of the cerebellum.^17^

Thus far, it appears that both in murine models of ASD and in autistic patients, anatomical and morphological changes in the cerebellum are common.^18^

## 2 NEUROIMAGING FINDINGS LINKED WITH ASD

### 2.1 Structural abnormalities

Neuroimaging studies have consistently reported structural abnormalities in the cerebellum of individuals with ASD. These abnormalities include reduced cerebellar volume^19^ and altered cerebellar morphology. For instance, the lobule VIIa‐Crus I and Crus II have been frequently associated with volume reductions in individuals with ASD.^12^ These regions are thought to be involved in higher order cognitive functions, such as attention, language processing, and social cognition, which are often impaired in individuals with ASD.

These findings suggest that structural aberrations in the cerebellum may be associated with the core features of ASD.

### 2.2 Functional abnormalities

Functional neuroimaging studies have also demonstrated atypical cerebellar activation patterns in individuals with ASD. Aberrant cerebellar connectivity with cortical regions involved in social cognition and language, such as the prefrontal cortex and the superior temporal gyrus, has been observed.^11^, ^20^ Moreover, disrupted cerebellar modulation of the default mode network, implicated in self‐referential thinking and social cognition, has been identified as a potential contributing factor to social difficulties in ASD.^21^, ^22^

## 3 NEUROBIOLOGICAL MECHANISMS AND FUNCTIONAL SIGNIFICANCE OF THE CEREBELLUM IN ASD

### 3.1 Cerebellar‐prefrontal circuitry

One proposed mechanism through which the cerebellum may influence ASD involves its connections with the prefrontal cortex. The cerebellum is known to modulate prefrontal cortical function, and disruptions in this cerebellar‐prefrontal circuitry could lead to deficits in executive functioning and social cognition seen in ASD.^12^, ^23^

### 3.2 Cerebellar GABAergic system

Alterations in the cerebellar GABAergic system have also been implicated in ASD. GABA, an inhibitory neurotransmitter, plays a crucial role in regulating neural excitation and inhibition. Studies have shown that GABAergic dysfunction in the cerebellum could disrupt the balance between excitation and inhibition in neural circuits, potentially contributing to the repetitive behaviors and sensory sensitivities seen in ASD.^6^, ^24^

### 3.3 Motor control and coordination

The cerebellum's traditional role in motor control and coordination is well‐established. While motor difficulties are not a core feature of ASD, many individuals with ASD exhibit fine and gross motor deficits.^25^ These motor impairments may be linked to cerebellar dysfunction, implicating the cerebellum in the broader motor and sensory aspects of ASD.

### 3.4 Cerebellar involvement in social and cognitive functions

Recent research has highlighted the cerebellum's involvement in social and cognitive functions, which are often impaired in individuals with ASD. Functional neuroimaging studies have shown cerebellar activation during tasks involving social cognition, theory of mind, and executive functions.^3^ These findings suggest that the cerebellum may contribute to the socio‐cognitive deficits observed in ASD.

### 3.5 Cerebellar modulation of cerebral function and developmental aberrations

The cerebellum may modulate cerebral activity, influencing various cognitive and emotional processes, and its dysfunction could lead to the cognitive and social impairments seen in ASD.^6^ Early cerebellar development and its interactions with the developing cortex may be perturbed in ASD, contributing to the emergence of the disorder.^5^

The expanded view of the cerebellum's function has led to significant advancements in understanding its modulation of cerebral function and its involvement in developmental aberrations. Recent research indicates that the cerebellum also plays a crucial role in cognitive processes such as attention, language, working memory, and executive functions. Functional neuroimaging studies have shown cerebellar activation during various cognitive tasks, suggesting its involvement in cognitive processing.^8^ Dysfunction in cerebellar circuits has been linked to emotional dysregulation and psychiatric disorders like depression and anxiety.

Emerging evidence suggests the cerebellum's contribution to social cognition, including theory of mind, empathy, and social decision‐making.^14^ Disruptions in cerebellar function may contribute to social impairments observed in conditions like ASD.^6^

Abnormal cerebellar development or function has been implicated in various neurodevelopmental disorders, including ASD. Genetic mutations affecting cerebellar development and function have been identified in individuals with neurodevelopmental disorders. These mutations often disrupt synaptic transmission, neuronal migration, or cerebellar circuit formation, leading to cognitive and behavioral impairments.^19^

The cerebellum exhibits remarkable neuroplasticity, particularly during development, allowing it to adapt to environmental inputs and optimize its connectivity. Disruptions in this process can result in developmental aberrations and contribute to the pathophysiology of neurodevelopmental disorders.^26^

## 4 ANATOMICAL ABNORMALITIES IN THE CEREBELLUM OF INDIVIDUALS WITH ASD

Several studies have reported structural abnormalities in the cerebellum of individuals with ASD. These abnormalities include reduced cerebellar volume^27^ and alterations in the morphology of cerebellar lobules.^6^ Notably, the cerebellum is disproportionately affected in individuals with ASD compared to typically developing individuals. These structural changes suggest a potential role for the cerebellum in the pathophysiology of ASD.

While ASD is typically associated with differences in brain structure and function, specific subtypes such as Rett syndrome and Angelman syndrome involve unique genetic or neurological mechanisms, often with implications for the cerebellum. Studies have suggested that individuals with Rett syndrome may exhibit abnormalities in the structure and function of the cerebellum. A study found reduced cerebellar volume in girls with Rett syndrome compared to typically developing girls, suggesting a possible link between cerebellar abnormalities and the motor impairments observed in Rett syndrome.^28^

While research on the cerebellar involvement in Angelman syndrome is somewhat limited, some studies have suggested cerebellar abnormalities in individuals with this condition. For instance, a study reported structural abnormalities in the cerebellum, including reduced cerebellar volume, in individuals with Angelman syndrome compared to typically developing controls, indicating a potential role of the cerebellum in the neurobiology of Angelman syndrome.^29^

### 4.1 Cerebellar‐cortical connectivity

The cerebellum is interconnected with various cortical regions, including the prefrontal cortex and posterior parietal cortex, which are known to be involved in higher order cognitive processes. Disruptions in cerebellar‐cortical connectivity have been observed in individuals with ASD,^12^ suggesting a potential mechanism for the cognitive and social impairments associated with the disorder.

### 4.2 Cerebellar‐cerebral connectivity

Alterations in cerebellar‐cerebral connectivity, particularly involving the default mode network (DMN), have been reported in individuals with ASD.^11^ The DMN is crucial for self‐referential thinking and social cognition, and its dysfunction has been implicated in ASD. Dysregulation of the DMN due to cerebellar abnormalities may contribute to the core features of ASD.

## 5 INVOLVEMENT OF CEREBELLAR LOBULES IN ASD

### 5.1 Lobule VI: Executive functions and language

One of the most intriguing findings in recent years is the involvement of cerebellar lobule VI in ASD. This lobule, traditionally associated with executive functions and language processing, has been consistently implicated in both structural and functional studies of individuals with ASD.^12^, ^30^ Moreover, disruptions in cerebro‐cerebellar connectivity involving lobule VI have been observed, suggesting its role in coordinating higher order cognitive processes affected in ASD.^31^

### 5.2 Lobule VIIb: Social cognition

Lobule VIIb, a region of the cerebellum implicated in social cognition, has also attracted attention in ASD research. This lobule is thought to play a critical role in understanding and interpreting social cues, which are often challenging for individuals with autism.^6^ Structural and functional abnormalities in lobule VIIb have been reported in ASD, suggesting a link between cerebellar dysfunction and social deficits.^5^, ^20^

### 5.3 Lobule Crus I and Crus II: Theory of mind

The theory of mind, the ability to attribute mental states to oneself and others, is often impaired in individuals with ASD. Lobule Crus I and Crus II of the cerebellum have been associated with theory of mind processes.^32^

**Figure 1:**
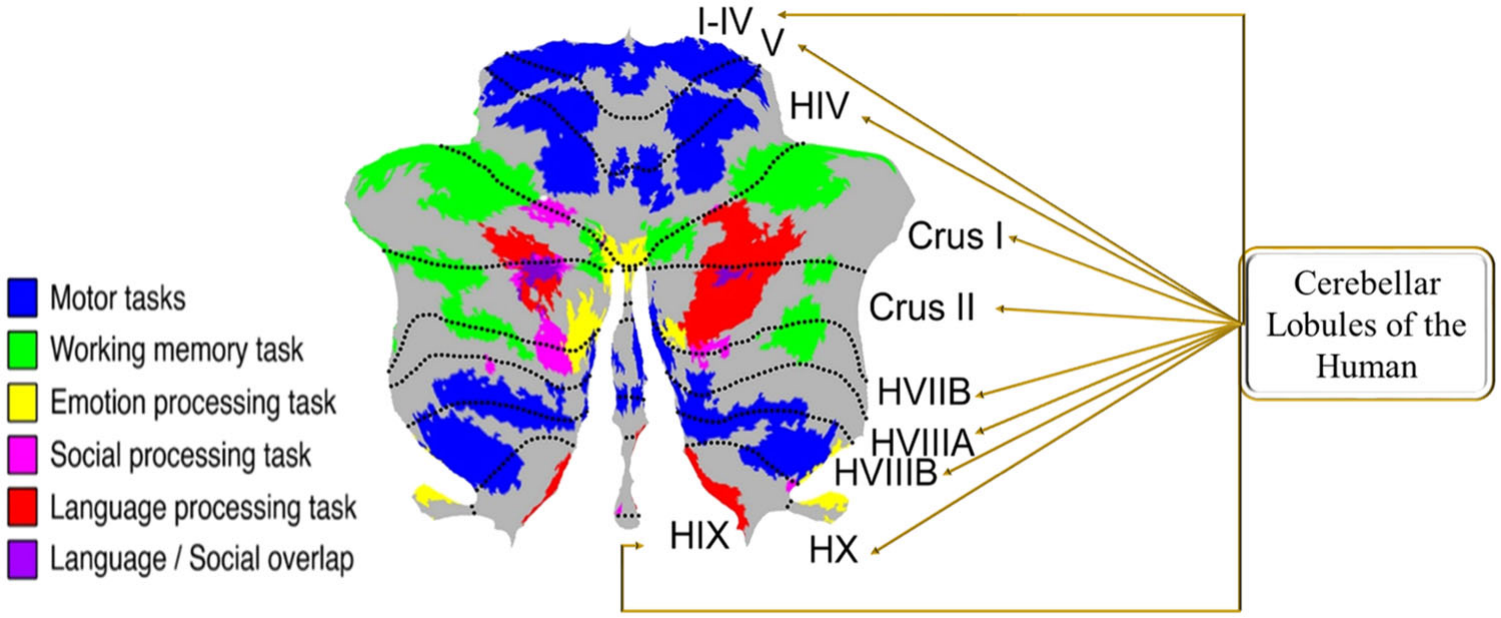
Illustrating the anatomical association of the cerebellum with executive function. Colored codes indicate the different functions of the cerebellar lobules. Figure modified from Guell et al.^33^

The anatomical association of the cerebellum with executive functions is very important to determine the ASD. Different lobules have different functions. Figure 1 shows the cerebellar lobules with their executive functions.

### 5.4 Potential mechanisms

While the exact mechanisms through which cerebellar lobules contribute to ASD remain to be fully elucidated, several hypotheses have emerged. These include:

Disrupted cerebro‐cerebellar connectivity: Abnormalities in the connections between the cerebellum and cerebral cortex may disrupt information processing and integration, leading to the characteristic symptoms of ASD.^12^

## 6 MECHANISMS UNDERLYING CEREBELLAR INVOLVEMENT

The exact mechanisms through which cerebellar lobules contribute to ASD pathophysiology remain under investigation. Some hypotheses suggest that cerebellar dysfunction could disrupt the coordination and timing of movements, speech, and social interactions, all of which are impaired in individuals with ASD. Other theories propose that cerebellar abnormalities may lead to atypical sensory processing, contributing to sensory sensitivities commonly observed in ASD. Additionally, the cerebellum is interconnected with several key brain regions involved in social cognition, and its dysfunction may disrupt the development of social skills.

The cerebellum's role in motor and cognitive functions: Understanding the cerebellum's involvement in ASD requires consideration of its broader role in motor and cognitive functions. The cerebellum is crucial for fine motor coordination, balance, and precise timing of movements.^13^ Furthermore, emerging research indicates that the cerebellum contributes to cognitive processes, including executive functions, working memory, and language.^14^

## 7 HOW CELLULAR MODELING OF CEREBELLAR STRUCTURE IS CONNECTED WITH DISEASE PATHOGENESIS RELATED TO ASD?

Cellular modeling of cerebellar structure can indeed offer valuable insights into the pathogenesis of diseases related to ASD. Understanding how abnormalities in cerebellar structure and function contribute to ASD can provide crucial insights into the underlying mechanisms of the disorder. Studies have shown that disruptions in the proper differentiation and migration of cerebellar cells during embryonic development can contribute to ASD. Cellular models allow researchers to study these processes in vitro, providing insights into how genetic and environmental factors may impact cerebellar development. For instance, induced pluripotent stem cells (iPSCs) derived from individuals with ASD can be differentiated into cerebellar cell types, allowing researchers to observe any aberrant differentiation or migration patterns.^34^

The cerebellum is rich in synapses and plays a crucial role in motor learning and coordination, as well as cognitive functions such as attention and social interaction. Dysfunction in synaptic function and plasticity in the cerebellum has been implicated in ASD. Cellular models allow researchers to investigate how genetic mutations associated with ASD affect synaptic transmission, plasticity, and connectivity within the cerebellum.^6^

Cellular models allow researchers to study gene expression profiles and molecular pathways specific to the cerebellum, providing insights into how genetic variants associated with ASD contribute to cerebellar dysfunction.^35^ Cellular models allow researchers to study the development and organization of cerebellar circuits, as well as how disruptions in these circuits contribute to ASD‐related behaviors.^36^

## 8 IMPLICATIONS FOR THERAPEUTIC INTERVENTIONS

Potential therapeutic implications: Given the growing evidence of cerebellar involvement in ASD, it is essential to explore potential therapeutic interventions that target this region. Noninvasive brain stimulation techniques, such as transcranial magnetic stimulation (TMS) and transcranial direct current stimulation (tDCS), have shown promise in modulating cerebellar function and may offer therapeutic avenues for individuals with ASD.^37^ Additionally, behavioral interventions that incorporate motor and cognitive training may help improve cerebellar function and mitigate some ASD symptoms.^25^

The recognition of cerebellar involvement in ASD has important clinical implications. First, it suggests that interventions targeting the cerebellum, such as cerebellar transcranial magnetic stimulation (TMS) or cerebellar‐specific therapies, may be considered as potential treatment options for individuals with ASD. Second, understanding cerebellar contributions to ASD can help develop more accurate diagnostic criteria and predictive models for the disorder, allowing for earlier and more precise interventions.

One aspect of cerebellar functional connectivity that has garnered attention is its potential involvement in E/I (excitation/inhibition) imbalance, which refers to an abnormal ratio between excitatory and inhibitory neurotransmission. Such imbalances have been implicated in various neuropsychiatric disorders, including ASD, schizophrenia, and attention‐deficit/hyperactivity disorder (ADHD).^38^

A study investigates cerebellar functional connectivity abnormalities in schizophrenia and psychosis‐risk individuals. While not explicitly focused on E/I imbalance, it sheds light on cerebellar involvement in neuropsychiatric disorders where E/I imbalance is implicated. Cerebro‐cerebellar connectivity abnormalities in ASD. Although not directly addressing E/I imbalance, it touches upon potential neurobiological mechanisms underlying ASD, some of which may involve disruptions in excitatory and inhibitory signaling.^36^

Another study focuses on functional connectivity in ADHD, it provides insights into how disruptions in neural circuitry, including those involving the cerebellum, may contribute to attentional deficits. Although not specifically addressing E/I imbalance, it highlights potential neural mechanisms underlying ADHD that may involve alterations in excitatory and inhibitory neurotransmission.^39^

Understanding the role of specific cerebellar lobules in ASD has several important implications. It opens new avenues for targeted therapies and interventions that focus on cerebellar function.

## 9 CONCLUSION

In conclusion, the cerebellum's role in ASD is increasingly recognized as crucial, with neuroimaging, neurobiological, and anatomical studies shedding light on its involvement. Neuroimaging studies have consistently shown alterations in cerebellar structure and function in individuals with ASD, suggesting a potential link between cerebellar abnormalities and the characteristic symptoms of the disorder.

Furthermore, neurobiological research has revealed disruptions in cerebellar circuitry and neurotransmitter systems, highlighting the complex interplay between the cerebellum and other brain regions implicated in ASD. Anatomical investigations have also provided evidence of cerebellar abnormalities, including changes in volume, morphology, and connectivity, further underscoring the significance of the cerebellum in ASD pathology. Specific cerebellar lobules appear to be implicated in various aspects of ASD, including social impairments, language deficits, and motor coordination problems. Overall, a comprehensive understanding of the cerebellum's role in ASD may offer new insights into the underlying mechanisms of the disorder and facilitate the development of more targeted interventions and treatments aimed at addressing cerebellar dysfunction in individuals with ASD.

## AUTHOR CONTRIBUTIONS

**Mohammad Shahangir Biswas**: Conceptualization; methodology; software; data curation; investigation; formal analysis; project administration; supervision; visualization; writing—original draft. **Suronjit Kumar Roy**: Investigation; software; data curation; writing—review and editing; formal analysis. **Rubait Hasan**: Writing—review and editing. **Md Moyen Uddin PK**: Writing—review and editing.

## CONFLICT OF INTEREST STATEMENT

The authors declare no conflict of interest.

## TRANSPARENCY STATEMENT

The lead author Mohammad Shahangir Biswas affirms that this manuscript is an honest, accurate, and transparent account of the study being reported; that no important aspects of the study have been omitted; and that any discrepancies from the study as planned (and, if relevant, registered) have been explained.

## Data Availability

All authors have read and approved the final version of the manuscript. [CORRESPONDING AUTHOR or MANUSCRIPT GUARANTOR] has full access to all of the data in this study and takes complete responsibility for the integrity of the data and the accuracy of the data analysis.
